# Chasing the ghost of infection past: identifying thresholds of change during the COVID-19 infection in Spain

**DOI:** 10.1017/S0950268820002782

**Published:** 2020-11-13

**Authors:** Luis Santamaría, Joaquín Hortal

**Affiliations:** 1 Estación Biológica de Doñana (EBD-CSIC), C/ Américo Vespucio 26, Isla de la Cartuja, E41092 Sevilla, Spain; 2 Department of Biogeography and Global Change, Museo Nacional de Ciencias Naturales (MNCN-CSIC), C/José Gutiérrez Abascal 2, 28006 Madrid, Spain

**Keywords:** Breakpoint regression, Catalonia, COVID-19, fatalities, growth curve, infection, Madrid, social distancing effectiveness

## Abstract

One of the largest nationwide bursts of the first COVID-19 outbreak occurred in Spain, where infection expanded in densely populated areas through March 2020. We analyse the cumulative growth curves of reported cases and deaths in all Spain and two highly populated regions, Madrid and Catalonia, identifying changes and sudden shifts in their exponential growth rate through segmented Poisson regressions. We associate these breakpoints with a timeline of key events and containment measures, and data on policy stringency and citizen mobility. Results were largely consistent for infections and deaths in all territories, showing four major shifts involving 19–71% reductions in growth rates originating from infections before 3 March and on 5–8, 10–12 and 14–18 March, but no identifiable effect of the strengthened lockdown of 29–30 March. Changes in stringency and mobility were only associated to the latter two shifts, evidencing an early deceleration in COVID-19 spread associated to personal hygiene and social distancing recommendations, followed by a stronger decrease when lockdown was enforced, leading to the contention of the outbreak by mid-April. This highlights the importance of combining public health communication strategies and hard confinement measures to contain epidemics.

## Introduction

COVID-19 infection has rapidly spread worldwide since its first outbreak in Wuhan (China) in mid-December 2019. The global number of confirmed cases has reached ca. 30 million on mid-September 2020 (John Hopkins University Coronavirus Resource Center [1]), 9 months after its first report on 31 December. Alike the 2002 SARS outbreak [2], individuals infected with SARS-CoV-2 remain asymptomatic for 5–6 days, while presenting enough viral load to be infective after 1–2 days of infection [3, 4]. Severe cases require hospitalisation 3–15 days after the appearance of the first symptoms, which are similar to other infectious respiratory illnesses. This, together with the initial unawareness of the population, led to a high transmission rate of the infection, which spread rapidly to neighbouring countries, the Middle East and Europe, and then the rest of the world (see https://nextstrain.org/ncov [5]).

An increasing number of countries was progressively affected, and they responded differently depending on the international (WHO) and local expert advice available at the moment, the structure and resources of their public health systems, their R&D capacity (which determined the number of PCRs available for testing contagions from swab samples, among other things) and their ability to implement social distancing measures [e.g. 6–9]. The diversity of policy responses, together with the pre-existing differences in spatial aggregation, social behaviour and age structure of their populations, provides a unique array of test cases to understand how different levels and combinations of preventive quarantine and social-distancing measures affected the spread of the pandemic.

COVID-19 arrived in mainland Spain in early February (first recorded hospitalisation dates back to 15 February), if not before [see 10]. During February, COVID-19 infection reached Spain repeatedly, mainly via the UK and Italy – as evidenced by the presence of 14 different genetic clusters identified by the Nextstrain project [5; accessed 27 April]. Different from Italy, where infections were concentrated in the North [9], the combination of these repeated introductions with early, unnoticed community transmission resulted in consecutive outbreaks in distant, highly populated areas of the Basque Country and Navarra (North), Madrid (Centre), Catalonia (North East), Andalusia (South) and Valencia (East). This is supported by preliminary evidence for an excess of cases diagnosed as influenza during February and March in Catalonia, compared with the historical record, which probably masked early COVID-19 infections [11]. The spatial structure of the Spanish populations played a role in the rapid spread of the pandemic in some regions the country [see 12]. Its impact was harsher in the large conurbations of Madrid (around 6.4 million people; second most populated Metropolitan area of the EU, after Paris) and Barcelona (c. 5.4 million), as well as in Álava, Navarra and La Rioja (c. 1 million in total) [13] following the early infection of healthcare workers from Txagorritxu Hospital. Balearic and Canary archipelagos also received infections from the early onset of the pandemic, so it is reasonable to assume that by early March COVID-19 infections were widely distributed throughout the whole country. The pandemic peaked, however, most strongly in Madrid – to the point that 65–67% of local incidence and mortality at the 52 Spanish provinces is explained by their early-stage mobility from and to Madrid [14].

Several factors support the use of Spanish data on the early expansion of COVID-19 to obtain a fair account of the effects of the pandemic at the country and regional levels. Alike Italy and South Korea, early records of the disease (until mid-April, see below) are unbiased, though incomplete. Although the lack of enough tests in the first months of the pandemic was pervasive for most countries (except South Korea), Spain achieved one of the highest testing ratios per capita at the time [15]. During this early period, only cases testing positive in the PCR make it to the official statistics, and (similar to Italy but different to other European countries) all deaths testing positive were registered as caused by COVID-19 infection, including those associated with previous pathologies. Until mid-April, these data excluded many deaths happening outside hospitals (e.g. in private homes and nursing homes) that had not been tested using PCR. From this date onwards, the nearly twofold increase in the testing capacity, as well as the inclusion of previous cases and deaths diagnosed from symptoms but not tested, created a serious unevenness in the time series. Although this update of cases provided a more realistic account of the extent of the epidemic, it created a reporting bias that hampers the fair comparison of data before and after mid-April [but see 12, 16].

Here we characterise the growth curve of COVID-19 infections in the whole of Spain, from the onset of the pandemic in early February through the establishment of increasing restrictions to mobility and personal contact. We also perform the analyses for the Madrid and Catalonia Autonomous Regions (Madrid and Catalonia hereafter), which represent the country's two largest foci of the pandemic. Madrid and Catalonia represent prime examples of the spread of the virus in large sets of populations (mostly panmictic for Madrid and spatially heterogeneous for Catalonia; see Methods) and the effect of social-distancing measures thereupon. The application of such social-distancing measures was broadly discussed by experts, media and social networks, with opinions ranging from qualifying them as exaggerated or unnecessary, during the first weeks of the outbreak; to criticizing them as tardy of insufficient, in the weeks that followed. Two controversies have been particularly strong: (i) were preventive and soft social-distancing measures useful, or should hard social-distancing measures have been introduced from the early moments (late February to early March)? (ii) did the mass events on the weekend of 7–8 March, coinciding with the International Women's Day demonstrations (over 300 K attendants in the whole country, of which 120 K in Madrid and 50 K in Barcelona) and premier football league matches (around 280 K spectators in total, of which 72 K in Madrid and 77 K and Barcelona) trigger the early spread of the pandemic in Spain's largest cities, especially in Madrid?

Bearing this temporal sequence in mind, we analyse the growth curves of the cumulative numbers of cases and deaths registered for the whole of Spain, Madrid and Catalonia, focusing specifically on the changes in the growth rate of these curves through time. Based on this analysis, we seek to answer two specific questions: (1) how effective were the different social-distancing measures in reducing infection and mortality rates? and (2) how significant were the effects of 7–8 March mass gatherings on the expansion of the epidemic, compared with other key events and control measures?

## Data and methods

### Timeline of events and control measures

Data on the different events that marked the evolution of the pandemic in Spain (e.g. first cases detected, large infection bouts, first deaths) or influenced its perception by the general public, as well as policy measures (e.g. preventive isolation, social-distancing, lockdowns) and putative key events (e.g. large gatherings associated to sport events, political demonstrations and party rallies), were gathered from official sources, national and international media, and scientific publications. Whenever possible, and in all cases for policy measures, we confirmed their date and content from official documents and/or websites from international, national or regional institutions. Owing to the discontinuity in detection effort and reporting procedures introduced by mid-April, we restrict our analyses to data from February, March and the first 2 weeks of April 2020. These data provide an underestimation of the total population infected and the number of fatalities, due to the limited number of tests, but the relatively homogeneous intensity of testing and the stability of criteria for disease attribution throughout time probably result in unbiased estimators for the spread of the pandemic. It is therefore safe to assume that the number of reported cases of infection and the number of deaths during this early period are reasonably good proxies for the advance of the pandemic.

### Infection and fatality data

Official data on the (i) cumulative number of cases, and (ii) cumulative number of deaths were obtained from the daily Covid reports of the Spanish Ministry of Health (first available at https://www.mscbs.gob.es/en/profesionales/saludPublica/ccayes/alertasActual/nCov/situacionActual.htm, and available now at the Centralized COVID Panel at https://cnecovid.isciii.es/covid19/). Data were extracted at two levels of aggregation, for Spain as a whole country, and for Madrid and Catalonia Autonomous Regions (i.e. *Comunidades Autónomas de Madrid* and *Catalunya*). Madrid is a highly populated area with good public transportation and a high daily commuting rate, while Catalonia combines Barcelona metropolitan area (the second largest in the country) with an extensive rural territory of low population density. Therefore, they represent prime examples of the spread of the virus in large sets of populations (mostly panmictic for Madrid and spatially heterogeneous for Catalonia) and the effect of social-distancing measures thereupon.

For the analyses, we included data from the first day in which at least 10 cases or at least one death were measured; and extended the analyses to 29–31 days after the onset of social-distancing measures on 13–15 March 2020, a period tripling the average infection-to-detection time (12 days; see next section), and almost doubling the average infection-to-death time (17 days; see next section). This ensures that the effects of these first measures are fully included in the dataset and also that, in the case of reported cases, the dataset covers the effects of the most stringent confinement measures starting the 31st of March.

### Lag time estimates

To estimate the infection date of reported cases, we calculated the infection-to-testing time by combining reported values of incubation time (mean = 5.0 days in [17]; median = 5.1 days in [3]; mean = 6.4 days in [4]) with time from illness onset to testing and hospital admission (median = 6 days, based on 158,094 diagnosed cases from Spain [12]). Hence, we used an infection-to-testing time of 12 days. Similarly, to estimate infection date from the day of death, we combined the reported values of incubation time (as above) and time from illness onset to death (median = 11 days, according to [12]) – which resulted in an infection-to-death time of 17 days.

### Analyses

We fitted a family of segmented (broken-line) regressions with no, one, two, three, four and up to five breaking points (Models 0–5, with two, four, six, eight, 10 and 12 parameters, respectively) and compared them using their respective AICs, using the segmented and lme4 packages of R 3.6.3 [18]. We chose the segmented package because, different to R's strucchange package [19] and other ‘structural breaks models’, it requires the fitted lines to join at the estimated breakpoints (i.e. it results in nearly-continuous models), which is consistent with the type of data analysed. However, we also conducted additional analyses using strucchange, to increase the probability of identifying additional breakpoints (see below). Models with a Relative Likelihood >0.05 were considered as equally good to that with the lowest AIC; we report primarily on the most parsimonious of these models (i.e. the model with less breaking points), but include also a brief discussion of all comparable models. Since we are analysing count data, the model with zero breaking points (and all segmented models built upon it) was fitted using Generalized Linear Models, with a Poisson error distribution and a log link.

For each model, we used the residual deviance and its degrees of freedom to calculate the scale (thus identifying residuals' overdispersion, if present) and evaluate model fit. While both values showed a marked improvement as model fit improved, they did not reach scale values significantly similar to 1 in all cases. Given the limitations of a strict use of the *P* values for the evaluation of model goodness-of-fit in Poisson regression [20], the strong improvement in both scale and test values in the better models (i.e. those with lower AICs), and the fact that increasing further the number of breaking points resulted in model instability, we are however confident that our models are as good as this technique allows.

Fitted breaking points provide objective information on the moment at which infection dynamics changed, while slopes provide information on the direction and magnitude of such changes. When analysing the data from Madrid, observed discontinuities suggested that some breakpoints could involve a change in the intercept, rather than in the slope. This would imply a significant shift in values at a given day, followed by a continuous increase at the same growth rate that preceded such day – a scenario consistent, for example, with a sudden increase in infection rate during the mass gatherings of 7–8 March. We test for this possibility using two approaches:

(1) We generated two sets of simulations, based on one of the data series (the number of cases from Madrid), and used them to evaluate the detectability of small changes in such variable, taking the events of 8 March as a key example. These simulations accounted for two different scenarios of increase in transmission rates associated to 7–8 March:

(i) A short-lived infection bout on 8 March 2020, as a 1-day increase in the number of cases of 25%, 50%, 75% and 100%, respectively. After this bout, the daily rate of increase observed in the data series was maintained for the remaining period (see Supplementary Fig. S4 for details).
(ii) A sustained increase in the infection rate from 8 to 15 March 2020 (the onset of the nationwide lockdown), as a rise in the daily rate of increase of 2.5%, 5%, 7.5% and 10%, respectively. After this, the daily rate of increase observed in the data series was maintained for the remaining period (as above; see Supplementary Fig. S5 for details).

Both simulations had comparable effects over the cumulative number of cases, as they resulted in increases of +25%, +50%, +75% and +100% on the last day of the data series. The simulated data series were then subject to the same analytical procedures applied to the reference dataset (segmented analyses with 1–5 breakpoints, followed by selection of the best model using AIC) and compared to it in terms of (i) the ability to detect a breakpoint on or near 20 August 2020 (corresponding to the day of infection bout plus the 12-day infection-to-detection period), and (ii) the magnitude of changes in all other breakpoints and slopes.

(2) We fitted a second set of segmented regressions using the breakpoints function of the strucchange R package [19] with a minimum of five data points per segment. This procedure is more sensitive to events in the early part of the curve. Further, while segmented uses an algorithm that minimises the increment in the fitted function at breakpoints (thus approaching continuity), strucchange uses separate intercepts at each different segment, thus allowing for discontinuities in the fitted functions – such as the ‘jumps’ and ‘stalls’ described above. This comes at a cost in terms of the increased number of parameters: one more per breakpoint (hence, models 1–5 have 5, 8, 11, 14 and 17 parameters, respectively), making the procedure more conservative in terms of breakpoint detection. Moreover, because this procedure can only be applied on ls models and we had to use log-transformed *y*-values to ensure linearity, the residuals showed considerable heteroscedasticity – with larger variance for smaller *x*-values. This means that the fitting procedure was more sensitive to variation in the lower part of the *x* (time) range, i.e. it tended to overestimate the number of breakpoints at the beginning of the curve and underestimate them at the end – as compared to the segmented fits. Altogether, the combination of both fitting procedures provides a more robust testing of the hypothesis presented above: breakpoints fitted in both procedures are likely to be the most relevant for the data series, and those detected by only one of the procedures are complementary in terms of the methods' sensitivity.

### Associations of disease growth with policy implementation and changes in citizen mobility

To explore further the potential associations between the temporal patterns of disease growth and the concurrent changes in policy and citizen mobility, we compared the breakpoints and segments derived from our six diagnostic variables (cases and deaths in Spain, Madrid and Catalonia) with two metrics. We measured governmental responses through the Stringency Index provided by Oxford Covid-19 Government Response Tracker [21]. We also explored the Containment & Health Index and the Economic Support Index provided there, but their high correlation with the SI for our sites and study period made them completely redundant. In the case of the changes to citizen mobility caused by these policies, we used a Mobility Index derived from Google's Mobility Report [22], calculated separately for each of the three spatial scales used (Spain, Madrid and Catalonia). We used the average of all (retail and recreation, grocery and pharmacy, parks, transit stations, and workplaces) but one (residential) mobility indices provided in such report. The Stringency Index data series was composed of a number of stable values, which changed abruptly (in 1–2 days) four times as a consequence of discrete policy measures. Hence, we could derive thresholds directly from the data. The data series of the Mobility Index, however, showed abrupt changes, as well as decreasing and increasing trends for some periods of time. To discern objectively the observed thresholds of change, we applied a segmented analysis similar to the one used for the six proxies of the pandemic (see above). For both variables (stringency and mobility), we analysed the association of detected thresholds with the breakpoints of disease growth (#cases and #deaths) by evaluating whether such thresholds fell within the 95% confidence intervals of the closest breakpoint.

## Results

### Timeline of events and policy measures

The adoption of containment measures by the national and regional governments followed a sustained increment through time, from (1) the recommendation of preventive measures in late February and early March; to (2) increasingly stricter social-distancing measures, which varied among regions, on 9–10 March; to (3) a nationwide lockdown announced on 13 March and enforced on 15 March; to (4) a strengthened lockdown, with the closure of all non-essential economic activities on 31 March (see Fig. 2 and Table S1). Non-essential economic activities were allowed again from 13 April, coinciding with the last value of the time series analysed here. Since infections are not detected as cases until several days later (see below), our data contain only a progressive strengthening of contention measures, without any de-escalation. A graphical summary with the timeline of the most relevant events and policy measures is presented in Figure 2 below (see Supplementary Fig. S1 for details), and a complete list of the events and measures compiled from our review is available in Supplementary Table S1.

### Number of cases

**Fig. 1.**
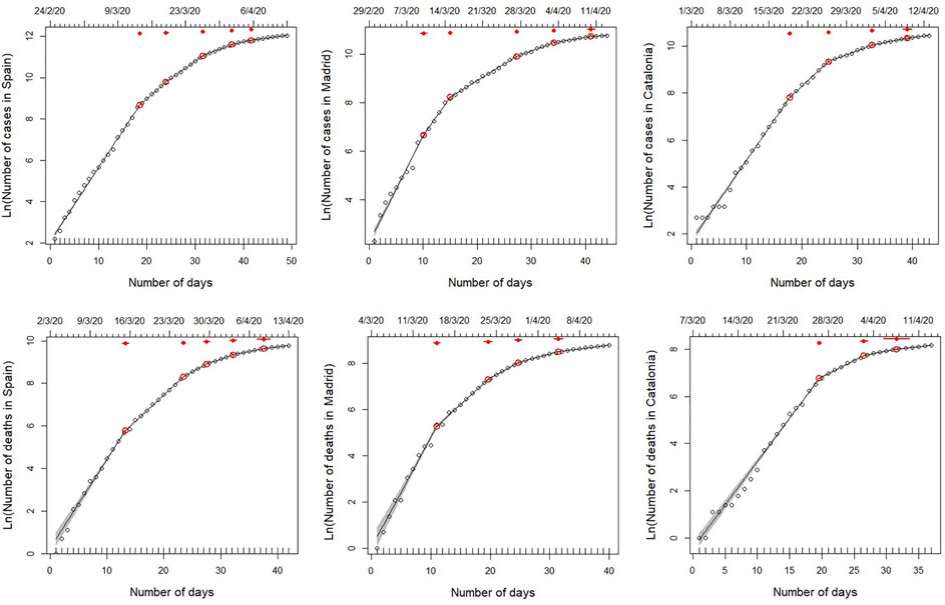
Segmented regressions fitted on the total number of cases detected in Spain and the two autonomous regions hosting its two largest cities (Madrid and Catalonia) from 25 February 2020 to 13 April 2020. Within this period, data series varies among variables and regions, since they start on the first day with >10 cases or >1 death (see upper *x*-axis for initial date). Lines show the best fit, as specified in Table 1 (see also Tables S2–S3). Red dots indicate breaking points of the best fit, with 95% confidence intervals (red lines). Note that, although the segmented analyses were performed on non-transformed data, we use a log-transformed *y*-axis for clarity.

For the whole of Spain, the model with five breaking points (Model 5) provided the best fit (Table 1). Fitted breaking points were placed on days 13–14, 19 and 26–27 March, and 1 and 5 April, corresponding to estimated infections on 1–2, 7, 14–15, 20 and 24 March (Fig. 1). The growth rate of the number of cases diminished by 42% after the first breakpoint, followed by decreases of 19%, 44%, 38% and 46% in the subsequent breaking points, respectively (see Table 1). The results from Madrid and Catalonia data series are largely consistent with those from the whole country, although some breakpoints occurred on different dates. In the case of Madrid, the model with five breaking points also provided the best fit (Table 1), with changes in trend on 10, 15 and 27 March, and 3 and 9 April (estimated infections on 27 February, and 3, 15, 22 and 28 March; Fig. 1). These breaking points are associated with decreases in the growth rate of 28%, 57%, 38%, 52% and 58%, respectively. Catalonia shows a slightly delayed, though quicker, pattern of disease containment, which was better fitted by the model with four breaking points (Table 1). Breakpoints occurred on 19 and 26 March, and 3 and 9 April (estimated infections on 7, 14, 22 and 28 March, respectively; Fig. 1), associated with progressive decreases of the growth rate of 36%, 59%, 49% and 37%, respectively.

**Table 1.** Results of segmented regressions with increasing numbers of breaking points, fitted on the total number of cases and the total number of deaths reported in Spain and the two autonomous regions hosting its two largest cities (Madrid and Catalonia) from 25 February 2020 to 13 April 2020

		#Cases in Spain	#Cases in Madrid	#Cases in Catalonia	#Deaths in Spain	#Deaths in Madrid	#Deaths in Catalonia
Best model		M5^a^	M5^a^	M4^a^	M5^a^	M4	M3
Intercepts	a1	2.064 ± 0.046	2.253 ± 0.095	1.704 ± 0.067	0.282 ± 0.173	0.039 ± 0.221	−0.947 ± 0.283
Slopes	b1	0.3571 ± 0.0029	0.4383 ± 0.0111	0.3438 ± 0.0045	0.4166 ± 0.0153	0.4755 ± 0.0230	0.4106 ± 0.0231
	b2	0.2059 ± 0.0032	0.3156 ± 0.0072	0.2196 ± 0.0027	0.2472 ± 0.0032	0.2349 ± 0.0066	0.3245 ± 0.0122
	b3	0.1669 ± 0.0009	0.1367 ± 0.0009	0.0893 ± 0.0012	0.1473 ± 0.0060	0.1440 ± 0.0069	0.1298 ± 0.0047
	b4	0.0936 ± 0.0011	0.0852 ± 0.0015	0.0459 ± 0.0015	0.0985 ± 0.0047	0.0705 ± 0.0030	0.0375 ± 0.0020
	b5	0.0584 ± 0.0009	0.0406 ± 0.0010	0.0291 ± 0.0017	0.0550 ± 0.0021	0.0325 ± 0.0017	
	b6	0.0314 ± 0.0004	0.0170 ± 0.0021		0.0346 ± 0.0024		
Breakpoints	u1	18.513 ± 0.102	10.046 ± 0.325	17.748 ± 0.180	13.192 ± 0.310	11.016 ± 0.291	14.640 ± 1.085
(#days)	u2	23.923 ± 0.261	15.015 ± 0.089	24.751 ± 0.085	23.417 ± 0.199	19.610 ± 0.350	20.318 ± 0.191
	u3	31.521 ± 0.065	27.104 ± 0.146	32.679 ± 0.169	27.359 ± 0.349	24.670 ± 0.312	27.422 ± 0.223
	u4	36.846 ± 0.119	33.625 ± 0.141	39.000 ± 0.420	31.714 ± 0.306	31.435 ± 0.386	
	u5	41.322 ± 0.114	40.168 ± 0.273		37.395 ± 0.493		
Goodness of fit^b^	*χ*^2^	284445.6	252.0	417.6	25796.5	11072.3	6568.4
	df	10	10	8	10	8	6
	*P*	<1.00E-300	2.10E-48	3.28E-85	<1.00E-300	<1.00E-300	<1.00E-300
Fit parameters	rDev	307.4	271.3	144.5	18.5	36.7	32.6
	df	37	32	33	30	30	29
	Scale	8.308	8.479	4.378	0.617	1.224	1.124
	*P*	2,48E-38	1,00E-39	6,70E-16	0.949618	0.1853	0.293924
	AIC	859.5	748.2	579.5	407.5	386.0	316.2
	BIC	882.2	769.6	593.1	444.9	402.9	329.0

Within this period, data series varies among variables and regions, since they start on the first day with >10 cases or >1 death. Therefore, start dates for each data series are 23 February 2020 for Spain, 1 March 2020 for Madrid, and 2 March 2020 for Catalonia in the case of cases, and 2 March, 4 March and 7 March for deaths. rDev: residual deviance.

a Fitted using seed breakpoint values.

b Poisson regression without breaking points as null model.

In general, the results of the segmented regressions fitted with the strucchange package support those described above (Supplementary Fig. S2). Both procedures consistently identified the 2–3 main breakpoints of each curve (i.e. 12–15, 19–20 and 25–29 March, corresponding to estimated infections on 29 February - 3 March, 7–8 March and 13–17 March) and showed comparable decreases in the growth rates at all of them (47–67%, 37–48% and 60–67%, respectively). As expected, strucchange failed to detect the two breaking points fitted by segmented at the end of the curve (on 30 March to 3 and 6–9 April), but it rather identified a significant increase in slope coupled to an increase in the intercepts (i.e. a ‘jump’ on the curve) on the early stages of the outbreak at Spain and Catalonia (on 4–7 March, estimated infection time on 23–26 February), but not in Madrid. This increase in growth rate was fairly small (19%) in Spain (where it was preceded by a larger decrease, of 37%, 1 week earlier), but very large in Catalonia (118%), where the start of the curve had been nearly flat until then. In Madrid, the apparent stall-and-jump of the number of cases visible on the two first weekends of March (28 February–1 March and 7–9 March 2020) did not result in any significant breakpoint in the strucchange fit.

### Number of fatalities

The number of fatalities associated with COVID-19 infection shows differences between Spain and the two regions, particularly Catalonia, where there were less breaking points but steeper changes in slope. The model with five breaking points (Model 5) provided the best fit (Table 1) for the whole of Spain. Changes in trend occurred on 15, 25 and 29 March, and 3 and 8 April, which correspond to patients estimated to become infected on 27 February, and 8, 12, 17 and 22 March (Fig. 1), and to decreases in the slope of 41%, 40%, 33%, 44% and 37% respectively. Results from Madrid were quite consistent with these breaking points, although in this case, the better fit corresponds to Model 4 (Table 1), with breaking points occurring on 15, 24 and 29 March, and 4 April, resulting from estimated infections on 27 February, and 7, 12 and 18 March, and associated to slope decreases of 51%, 39%, 51% and by 54%, respectively. Although the curve of accumulated fatalities in Catalonia showed a lower number of segments (Model 3 provided the best fit; Table 1), the dates of the breakpoints coincided with those detected in Madrid and Spain: 22 and 27 March, and 3 April, resulting from estimated infections on 5, 10 and 17 March (Fig. 1) and associated to slope decreases of 21%, 60% and 71%.

The results of the segmented regressions fitted with the strucchange package generally supported those described above. Both procedures consistently identified the 2–3 main breakpoints of each curve (i.e. 12–15 and 25–29 March, and 2–4 April, corresponding to estimated infections on 24–27 February, 8–12 and 16–18 March) and showed comparable, albeit lower, decreases in the growth rates at all of them (41–43%, 39–60% and 44–72%, respectively). As expected, strucchange identified one additional breakpoint at the beginning of each of the three curves (on 7–12 March, estimated infection time on 19–24 February), while failing to detect approximately half of them (four out of nine) at the end of the three curves. The two early breaking points detected on 12–13 and 17–18 March in Catalonia corresponded to changes in the intercepts (a ‘dive’ and a ‘jump’ in values, both from one day to the next), with virtually no change in the slopes. In Madrid, two similar jumps detected on 6–7 and 12–13 March (estimated infections on 18–19 and 24–25 February) were associated in contrast with considerable decreases (32–43%) in the growth rate.

### Associations between breakpoints and policy events

**Fig. 2.**
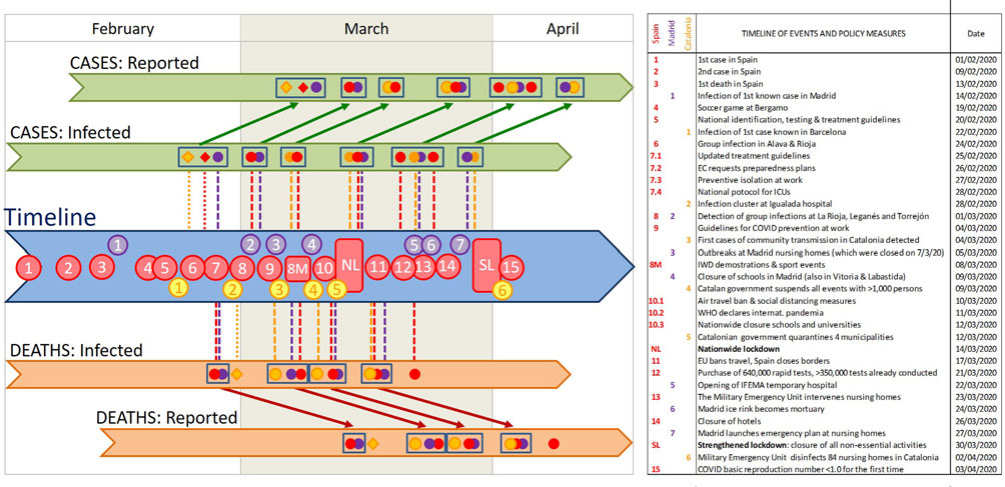
Timeline of the key events for the spread of COVID-19 in Spain, the increased awareness of the Spanish population, and Control Measures taken by the government. Coloured dots indicate the breaking points identified in our segmented analyses for the whole of Spain (red) and specifically for Madrid (purple) and Catalonia (yellow). Diamonds indicate sudden increases in the intercept, identified as breaking points by the strucchange analysis. Clusters of breaking points are identified with rectangles. The position of these breaking points in the 'reported' sections indicates the date detected in our analyses of the temporal COVID-19 growth curves, and those in the 'infected'sections indicate the estimated date of infection. Numbers in the timeline indicate the key events listed in the table. Vertical lines indicate potential synchronies between events in the timeline and estimated changes in the growth rates (i.e. breaking points) of both cases and deaths (above and below the timeline, respectively). Diagonal arrows link the estimated infection dates and the detection dates of identified breaking points, for both cases and deaths. Note that the combination of vertical and diagonal lines indicates the dates at which changes in infection dynamics could be perceived, for the first time, as breaking points in the numbers of cases or deaths. WMC stands for World Mobile Congress, and EC for European Commission. See Supplementary Table S1 for a detailed account of the timeline, and Supplementary Figure S1 for an account including the variation in the growth rate (slope of the ln-transformed data) at each breaking point.

The results of the segmented regressions show a consistent temporal pattern of disease growth that can be divided into six consecutive phases linked to a series of events and policy measures (Fig. 2).

(1) A first phase, in the early moments of the epidemic in Spain, characterised by fast and sometimes sudden increases in the number of infections (i.e. ‘jumps’). These jumps are particularly conspicuous in Catalonia and Madrid for the number of cases (infections from 23 to 27 February) and in Spain for the number of fatalities (infections from 29 February). This phase coincided with the detection of the first cases, imported from abroad; and the two jumps in the number of infections were synchronous with specific events of group infections (at sport events, first, and hospitals, nursing homes and churches, later; red point 4 and yellow points 1–2 in Fig. 2; see also Supplementary Table S1 and Supplementary Fig. S1). Owing to the lag times mentioned above, the effects of this phase become perceivable between 6–8 and 17 March (for cases and deaths, respectively).
(2) Overlapping with the above-mentioned increases and closely following them, there is a second phase characterised by strong reductions in the numbers of cases and deaths at two of the three areas (Spain and Madrid). Four different breaking points observed on 14–15 March 2020 indicate 42–57% decreases in the slope of the number of cases (estimated infections on 2–3 March 2020) and 41–51% decreases in the slope of the number of deaths (estimated infections on 27 February 2020; Fig. 1). These changes are probably associated to the improvement of clinical procedures (detection and hospital treatment) following the issuing of specific guidelines for the identification, treatment and research of Covid19 cases by the Spanish Ministry of Health and the regional authorities (issued on 19–20 February 2020, updated on 25 February 2020) and the first preventive regulations (e.g. for preventive isolation at work, on 27 February 2020). Strikingly, the perception of strong slowdown in 14–15 March, broadly understood in the Spanish media as a reflection of the success of the social distancing measures, was actually caused by the combined impact of these two sets of previous events: the reduction in the number of infections starting on 2–3 March (phase 2), and the reduction in the number of deaths starting on 27 February (phase 1) – underscoring the decoupling of cause and perception (see below).
(3) A third phase showing a highly consistent pattern of decreases in the growth rates of both the number of cases and the number of fatalities at the three geographical extents (49–57% and 21–40% reductions for cases and deaths, respectively), corresponding to infections starting on 5–8 March. This cluster of breakpoints is the second most consistent and supported, coinciding closely in time for five out of six of the time series evaluated. It precedes the issuing of the most stringent social-distancing measures by the central and regional governments (‘NL’ in Fig. 2), but follows on from the issuing of preventive isolation measures by the Labor Ministry, guidelines for COVID-19 treatment at hospitals and Intensive Care Units by the Health Ministry, and other measures by central and regional agencies (red points 7 and 9 in Fig. 2). Owing to the lag times mentioned above, these decreases become perceivable between 19 (for the number of cases) and 22–25 (for the number of deaths) March.
(4) A fourth phase, starting only a few days later, showing a consistent set of reductions for the slope of the number of deaths (for Spain, Madrid and Catalonia). This cluster of breaking points corresponds to infections on 10–12 March, and resulted in strong slope reductions (33–60%). The most likely cause is the issuing of partial social-distancing measures by the central and regional governments (purple point 4 and yellow point 4 in Fig. 2) and, at national level, the issuing of increasingly stringent measures such as an air travel ban and the closure of schools and universities (red point 10 in Fig. 2).
(5) A fifth phase, starting 5–10 days later, showing a consistent pattern of decrease in the growth rates of the number of cases (38–59% decrease, estimated infections on 14–15 March 2020) and the number of deaths (44–71% decrease, estimated infections on 17–18 March 2020) at the three spatial extents (Spain, Madrid and Catalonia). This cluster of breakpoints is the most consistent and supported, coinciding for all six growth curves and present in all models with two or more breakpoints, in most models with one breakpoint, and in the alternative fits using strucchange. This indicates that this was the most important change in infection dynamics during the period of time analysed. It coincides closely with the issuing of strong social-distancing measures (nationwide lockdown and border closure on 13–14 March 2020), albeit showing a small (3–4 days) delay for the number of deaths. The impact of this sharp change on infection trend was perceivable on 26–27 May and 3–4 April (for the number of cases and deaths, respectively).
(6) Two additional clusters of breakpoints for the number of cases, caused by reductions in the number of infections starting on 20–24 and 28 April, which do not seem directly related to any specific event or policy measure – and is most likely related to the strengthening of the lockdown as it was being progressively enforced at local levels (as reflected in the progressive decrease in mobility, see next section).

In contrast with other policy measures, which resulted with highly consistent clusters of breaking points at the three spatial levels of analysis, the strengthened lockdown issued on 30 March had no perceivable effect in the number of cases for any of the spatial extents analysed. Unfortunately, the data series is not prolonged enough to allow for a robust evaluation of the effect of the strengthened lockdown on the number of deaths.

### Simulations of increases in growth rate associated with mass gatherings on 7–8 March

The segmented analyses of the two sets of simulated datasets indicated that this procedure would have a high sensitivity to detect changes associated with the two types of increases in growth rate (a 1-day infection bout and a 1-week increase in infection rate) introduced in the data. All simulations but one (the 2.5% rise in the rate of increase) resulted in the detection of a breakpoint on 20–23 March 2020, which was not detected in the original dataset (Supplementary Fig. S4 and S5). The two types of increases had, however, different effects on the results: while the sustained increase in the infection rate resulted in an increase in the slope of the segment after the breakpoint, the infection bout resulted in the opposite (a decrease in the slope after the breakpoint). These results confirm that our analyses would have detected any significant change of trend, sustained or not, in COVID-19 spread rates associated with mass gatherings on the weekend of 7–8 March. They also suggest, however, that the first breakpoint detected in Madrid for numbers of cases and deaths (10 and 15 March, corresponding to infections on 27 February) may be a result of the distinct jump in values 1–2 days before.

### Associations of disease growth with policy Stringency Index and citizen mobility

The analyses of the Mobility Index show a large and abrupt decrease over a period of 3–4 days and a small, slow decrease afterwards (Supplementary Fig. S6). This pattern is consistent for the whole country and the two regions. The first, main decrease is tightly associated to the abrupt increase in Stringency Index on 13–15 March 2020 (nationwide lockdown and border closure): it started 1–2 days before its official declaration (11–12 March 2020) and finished right after its enforcement (15–16 March 2020). After this point, mobility values kept decreasing until the end of the data series analysed here, albeit at a much lower rate; and for one of the regions (Catalonia), there is a second breakpoint associated to an additional decrease in the slope on 22 March 2020.

The thresholds in Stringency Index and Mobility Index were closely associated to breakpoints in the number of cases and/or the number of deaths at the three geographical extents examined (Spain, Madrid and Catalonia; Table 2). Breakpoints in the number of cases and/or deaths corresponding to infection dates taking place after 10 March 2020 were thus associated with the continuous decrease in mobility found in such period. However, the early breakpoints detected at the beginning of the time series (before 9 March 2020) were not associated to any reported changes in stringency or mobility – reinforcing the conclusion that they were probably related to other factors (see the previous section).

**Table 2.** Association between breakpoints in disease growth dynamics (total number of cases and deaths) and thresholds of change in policy stringency and citizen mobility, reported in Spain and the two autonomous regions hosting its two largest cities (Madrid and Catalonia)

Spain															
Cases				Deaths				Stringency index				Mobility index			
% change	Detection date	Infection date	CI95%(days)	% change	Detection date	Infection date	CI95%(days)	Value	% change	Date	Day	Value	% change	Date	CI95%(days)
−42.3%	13–14/03/2020	01–02/03/2020	61.4–61.7	−40.7%	15/03/2020	27/02/2020	57.6–58.8								
−18.9%	19/03/2020	07/03/2020	66.4–67.4	−40.4%	25/03/2020	08/03/2020	68.0–68.8								
								45.8	83.3%	9–10/03/2020	70.0				
				−33.1%	29/03/2020	12/03/2020	71.6–73.1					−1.2%		12/03/2020	71.9–72.9
−43.9%	26–27/03/2020	14–15/03/2020	74.5–74.7					67.1	46.5%	14/03/2020	74.5	−66.2%	−65.0%	15/03/2020	74.3–75.6
				−44.2%	03/04/2020	17/03/2020	76.1–77.3	71.8	4.0%	16–17/03/2020	77.0				
−37.6%	01/04/2020	20/03/2020	80.7–81.0												
				−37.1%	08/04/2020	22/03/2020	81.4–83.4								
−46.2%	05/04/2020	24/03/2020	87.0–87.6												

Madrid															
Cases				Deaths				Stringency index				Mobility index			
% change	Detection	Infection	CI95%(days)	% change	Detection	Infection	CI95%(days)	Value	% change	Date	Day	Value	% change	Date	CI95%(days)
−28.0%	10/03/2020	27/02/2020	57.4–58.7	−50.6%	15/03/2020	27/02/2020	57.4–58.6								
−56.7%	15/03/2020	03/03/2020	62.8–63.2												
				−38.7%	24/03/2020	07/03/2020	65.9–67.3								
				−51.0%	29/03/2020	12/03/2020	71.0–72.3	45.8	83.3%	9–10/03/2020	70.0	−2.2%		11/03/2020	70.7–71.8
−37.7%	27/03/2020	15/03/2020	74.8–75.4					67.1	46.5%	14/03/2020	74.5	−68.8%	−66.6%	15/03/2020	74.6–75.5
				−53.9%	04/04/2020	18/03/2020	77.6–79.2	71.8	4.0%	16–17/03/2020	77.0				
−52.3%	02–03/04/2020	21–22/03/20	81.3–81.9												
−58.1%	09/04/2020	28/03/2020	87.6–88.7												

Catalonia															
Cases				Deaths				Stringency index				Mobility index			
% change	Detection	Infection	CI95%(days)	% change	Detection	Infection		SI	% change	Date	Day	Value	% change	Date	CI95%(days)
				−21.0%	22/03/2020	05/03/2020	62.4–66.9								
−36.1%	19/03/2020	07/03/2020	66.4–67.1												
				−60.0%	27/03/2020	10/03/2020	69.9–70.7	45.8	83.3%	9–10/03/2020	70.0				
												1.2%		12/03/2020	71.9–72.8
−59.3%	26/03/2020	14/03/2020	73.6–73.9					67.1	46.5%	14/03/2020	74.5	−64.6%	−64.6%	15/03/2020	74.2–75.2
−48.6%	03/04/2020	22/03/2020	81.3–82.0	−71.1%	03/04/2020	17/03/2020	77.0–77.9	71.8	4.0%	16–17/03/2020	77.0	−81.6%	−17.0%	22/03/2020	77.3–86.7
−36.6%	09/04/2020	28/03/2020	87.1–88.8												

Each row includes all associated breakpoints and thresholds. Note that, to allow for a comparison of values across the different variables, 95% CI are expressed as day of the year (with 1 January 2020 being day 0) and, for the #cases and #deaths, calculated for the estimated date of infection.

## Discussion

Our analysis of the growth rates of cases and fatalities evidences the effectiveness of the contention measures taken by Spanish national and regional governments. The results of the six variables analysed, involving three different spatial settings and two different lag times, show a consistent temporal pattern that can be divided into six coherent phases. The phases are associated with the gradual implementation of increasingly stringent measures and policies (*sensu* [21]), as well as to the issuing public awareness and public health recommendations [23]. After 9–10 March, the confinement of all population that could telework, the suspension of in-class teaching in schools and universities, and the closure of non-essential shops, bars and restaurants resulted in marked downward inflections in all the curves of infections and deaths. More remarkable is, however, the existence of two earlier clusters of breakpoints, corresponding to decreases in the rates of infections and deaths in the last week of February and the first week of March. This first dropdown in COVID-19 growth rates precedes the legal enforcement of most social-distancing measures by the regional and central governments. Indeed, it takes place the 2 weeks before the marked decrease in community mobility registered during the second week of March (from 8 to 15 March) in Madrid, Catalonia and the whole of Spain [22]. Instead, it coincides with the issuing of official recommendations for the prevention and treatment of COVID infections, and an increase of the awareness of the Spanish population (probably due to the reporting of a rapidly increasing number of cases and deaths).

The existence of significant diminutions in the disease growth rate before the enforcement of stringent movement-restriction policies suggests that a large proportion of the Spanish population changed their behaviour due to the combination of increasing awareness and the dissemination of preventive hygienic and distancing measures by the government and the media – with recourse to the examples of China, South Korea and Italy. Personal hygiene has been instrumental to the long-term success of public health policies [24] and public health campaigns directed to increase personal hygiene can successfully prevent disease incidence in the population [25]. Indeed, training in personal hygiene and self-protective behaviour helped protecting health care workers and the general population during SARS outbreaks [2, 26, 27]. Hence, an outreach strategy that increases awareness in the general public and disseminates good practice in personal hygiene may have significant effects on the spread of airborne diseases such as COVID-19. In the case of Spain, it seems fair to argue that during the second week of March (8–15), the population responded swiftly to the voluntary prevention and distancing measures suggested by the authorities and the media [23] and these were effective to slow down the early spread of the pandemic. Interestingly, a similar slowdown of COVID-19 growth rates before the enforcement of the population lockdown was not observed in China [28], probably due to the initial lack of information about the potential for easy transmission of the disease. The different response of the Spanish population provides evidence for both the potential effectiveness of controlling the initial growth of the pandemic through ‘soft’ contention measures based on public health policies and an enhanced use of the primary health care system (as advocated by [29]) and the key importance of media participation for the success of these soft measures [30, 31].

The results also indicate, however, that these measures were not enough to contain the spread of the virus – and the issuing of soft social-distancing measures by the regional governments had only a moderate impact upon it (phase 3). The next breaking point, resulting in the strongest reduction in the growth rate of the numbers of cases and deaths, took place closely after the onset of the national lockdown and border closure. As in the previous phase, the population's response was swift and started already with the partial confinement measures, 1 week before [e.g. 22] – greatly surpassing the expectations of 69 experts who predicted a collapse of the health system on 25 March [32–34]. These stronger isolation policies were probably instrumental in flattening the curve of infections, though not sufficient to bring it to a complete halt. Similar to this case, strong lockdowns involving the enforcement of stringent restrictions to personal movements, use of public spaces, non-essential economic activities and in-office work have successfully contained the spread of COVID-19 in many countries and regions [28, 35–40]. Indeed, comparisons between related countries evidence that stricter lockdown policies reduce the number of COVID-19 deaths [41]. Their success has been however affected by the tensions between national, regional and local governments, to the point of being jeopardised when different administrations and political parties were unable (or unwilling) to coordinate efforts [42]. Indeed, the examples of France and Spain indicate that the implementation of the lockdown resulted in massive reductions of short-range mobility, but the lack of coordination between administrations resulted also in anomalous increases of long-distance movements between regions [14, 43]. This is particularly unfortunate since lockdowns impose significant difficulties for poor households and food security [e.g. 44], so their use must be necessarily limited in time.

What our results fail to show are the expected effects of any of the key events pinpointed by the media on the spread of COVID-19 infections. The demonstrations and football matches of 7–8 March did not result in significant increases in neither infection rates nor the number of infections (as reflected in the numbers of cases and fatalities). The public perception that these events may have boosted transmission rates is inconsistent with both the observed patterns of numbers of cases and deaths in the three spatial extents analysed, and our simulations of anomalous increments in disease growth rates in Madrid. This misperception probably arose from the lag times between infection and case report (see below), as the eventual effects of these mass gatherings would have shown up 12–17 days later, not immediately after it. Strikingly, the only breakpoints that could be consistent with a change in the number of infections on 8 March (19 March for the number of cases in Spain and Catalonia, and 24–25 March for the number of deaths in Spain and Madrid) show a decrease in the infection rates, rather than the expected increase in either the slope or the intercept. The stalling-and-jump pattern observed the 7–8 March weekend, which can also be observed in the previous and following weekends (27 February and 15 March, for the number of cases and the number of deaths, respectively), is probably related to earlier infection bouts (see Fig. 2) or to reporting errors (reduced case and fatality reporting during the weekend, with a subsequent increase on Monday–Tuesday).

It is also apparent that the prolonged infection-to-detection lags at the early stages of COVID-19 spread may have caused misperceptions of both the effectiveness of certain measures and the impact of certain events (most notably, the 8th March demonstrations and several sport events), but were later instrumental in triggering the widespread social response during phase 2 of the outbreak in Spain. With hindsight, it is clear that issuing strong social-distancing measures earlier would have increased their effectiveness, thereby saving more lives and reducing the collapse of the Spanish health system (see e.g. the simulations of [45] for Spain or [46] for the USA). Indeed, the early response of Portugal, which rapidly followed up the lockdown and mobility restrictions enforced by the Spanish government within a few days, was instrumental in its success to contain the initial wave of the pandemic [47]. The geographic distribution of seroprevalence [13] evidences that many Spanish regions had similar success, benefiting from a nationwide lockdown whose imposition was based on the situation in a handful of severely affected regions (Madrid, Catalonia, La Rioja and the Basque Country). Our analyses evidence, however, that the responses of the population, media and authorities were slowed down by the perceptual trap created by COVID-19's prolonged infection-to-detection and infection-to-death lags. It is somehow ironic that the rapid increase in the infections during the last weeks of February (described in phase 1) was only perceivable 2 weeks later – and, while the increments in the number of cases and deaths were attributed to concurrent events that were most likely unrelated, they triggered a swift response precisely at a moment when early containment measures were already starting to work. Similarly, the results of early containment measures were perceived 2 weeks later, and attributed to a direct consequence of the national lockdown. Fortunately, expert advice to the government was undoubtedly aware of this perceptual trap and insisted on the necessity of stronger social-distancing measures – which were narrowly sufficient to reach the objective of flattening the curves by mid-April, as predicted by [48].

Our results provide a preliminary demonstration that most of the increasingly stringent policies that were progressively implemented in Spain during March 2020 were successful to reduce COVID-19 growth rates, from the first personal hygiene recommendations to the hard nationwide lockdown. In contrast, the most stringent measure implemented (the ban on all non-essential economic activities) showed an indiscernible success. A systematic evaluation of the implementation of public health policies and measures [49] during SARS-CoV-2 outbreak would be required to ascertain the value of each one of the enforced policies, in Spain as well as other countries. The use of segmented regressions can provide an objective procedure to identify thresholds of change during the evolution of COVID-19 pandemic (see [16]), and therefore be useful for these systematic evaluations (see also [38, 39, 50]).

To summarise, the patterns of change uncovered by our analyses show that a combination of public awareness, personal hygiene and social distancing can help slow down COVID-19 spread in future outbreaks – such as the one already ongoing in Spain or Israel in September 2020 (see also [29]). These measures should however be accompanied by policies that were not initially implemented but have been successful to contain SARS-CoV-2 and other SARS and MERS outbreaks [51], including wearing face masks [52], digitally-enhanced social distancing measures [53, 54], selective school and university closures [50, 55], active strategies for tracing contacts and selective quarantines [2, 26], as well as performing engineering controls and adapting indoor spaces to enhance ventilation in public buildings [56]. The implementation of these policies should be accompanied by a more effective communication strategy that helps engaging the general public into following measures whose effectiveness can be misperceived due to the long infection-to-detection lags of SARS-CoV-2 infections. This virtuous combination of active public health and information policies, and the reinforcement of primary health care would help avoiding the harsh economic and social consequences of having to recourse to hard lockdowns in current and future outbursts of COVID-19 and other pandemics.

## Supplementary material

For supplementary material accompanying this paper visit https://doi.org/10.1017/S0950268820002782. click here to view supplementary material
