# Supplementary material for: Chasing the ghost of infection past: identifying thresholds of change during the COVID-19 infection in Spain
Source: Epidemiol Infect. 2020 Nov 13;148:e282. doi: 10.1017/S0950268820002782 (PMC7729171; doi:10.1017/S0950268820002782)

**Epidemiology and Infection, Chasing the ghost of infection past: identifying thresholds of change during the COVID-19 infection in Spain, Santamaría & Hortal**

**Supplementary Materials**

**Supplementary Table S1.** Detailed timeline of the outbreak and management measures in Spain. Based on Ravelo & Jerving (2020) plus local sources.

| **Date** | **Outbreak dynamics** | **Key events & management measures** |
| --- | --- | --- |
| 28/1/20 |  | The Spanish Ministry of Health publishes a protocol for the management of potential COVID cases, including 14-days quarantine following contact with infected individuals. |
| 31/1/20 | First case detected in Spain: a German tourist in Canary Islands | Iberia cancels all flights to China. Spanish from Wuhan quarantined in Madrid.  Arrival of Wuhan Zall football team to Málaga for a training camp in Cádiz. |
| 9/2/20 | Second case detected in Spain: an English tourist in Mallorca |  |
| 12/2/20 |  | World Mobile Congress cancelled in Barcelona |
| 13/2/20 | First deceased in Valencia, will be detected in necropsy on 4/3/20 |  |
| 14/2/20 | Latest possible infection of a woman from Torrejón (Madrid) who travelled to Ecuador on 14/2 and developed her first symptoms on 16/2 |  |
| 15/2/20 | One case hospitalized in Madrid, will be tested and detected on 28/2/20 |  |
| 19/2/20 |  | 2,500 Valencia soccer fans mixed with 40,000 Atalanta supporters for a Champions League game in Bergamo |
| 20-21/2/20 |  | National guidelines for the identification, sampling/testing, registration and treatment of COVID cases, distributed by regional governments |
| 22/2/20 | Return to Barcelona of a tourist who got infected in Italy, will be tested and confirmed on 24/2/20 |  |
| 23/2/20 | Italy becomes largest outbreak outside Asia | Guidelines on the [prevention and control during the management of COVID patients](https://www.mscbs.gob.es/profesionales/saludPublica/ccayes/alertasActual/nCov-China/documentos/Documento_Control_Infeccion.pdf) published by the Ministry of Health |
| 24/2/20 | 3rd case detected, an Italian doctor spending holiday in Tenerife.  Largest infection event known in Spain: 60 persons from La Rioja and Alava get infected during a funeral. Cases will be detected on 7/4/20. |  |
| 25/2/20 | First cases in inland Spain, at Madrid, Barcelona and Castellón  First school teacher taking sick leave , will be hospitalized on 1/3/20 | Updated guidelines for the identification, sampling/testing , registration and treatment of COVID cases distributed by regional governments |
| 26/2/20 | First reported case in Andalusia | *The EC requests member states to review pandemic preparedness plans and inform back on how they plan to implement them* |
| 27/2/20 | 8 new cases of Italian/Iranian origin in Catalonia, Castilla-León & Valencia.  In Valencia, 4 out of 6 new cases were infected by a sport journalist who attended Bergamo on 19/2/20, got sick on 25/2/20 and hospitalized on 27/2/20 |  |
| 28/2/20 | 9 new cases in Andalusia and 1 more in Madrid (already hospitalized but not tested on 15/2/20).  Infection cluster at Igualada, including a large group of health professionals from Igualada hospital | **Preventive isolation measures** for SARS-CoV-2 (quarantine) will be considered as temporary incapacity by Social Security  1^st^ version of the [Protocol for the clinical management of COVID at ICUs](https://www.mscbs.gob.es/en/profesionales/saludPublica/ccayes/alertasActual/nCov-China/documentos/Protocolo_manejo_clinico_uci_COVID-19.pdf), hospitals and primary care published by the Ministry of Health, updated on 9/3/20 and 19/3/20 |
| 1-2/3/20 | Infection of the group of 60 persons from La Rioja and Alava during a funeral gets detected  Group infection of 7 persons at a Christian church in Leganés (Madrid) and cluster of 6 local infections at Torrejón de Ardoz (Madrid) |  |
| 3/3/20 | 150 cases, of which 49 in Madrid, 15 in Catalonia and Valencia, 13 in Basque Country. First death in Madrid, not confirmed until 5 March | Government announces intention to declare moderate social-distancing measures in Madrid and Basque Country |
| 4/3/20 | First cases of community transmission detected in Catalonia | **Soft social-distancing measures:** The Labour Ministry publishes [guidelines for COVID prevention at work,](http://www.mitramiss.gob.es/ficheros/ministerio/inicio_destacados/Gua_Definitiva.pdf) including temporary cessation of activity (complete guide [here](https://www.mscbs.gob.es/profesionales/saludPublica/ccayes/alertasActual/nCov-China/documentos/Procedimiento_servicios_prevencion_riesgos_laborales_COVID-19.pdf)). |
| 5/3/20 | 10 persons infected in a nursing home for the elderly, following the death of one person on 3/3/20.  1 dead & 20 infected in a nursing home at Valdemoro (Madrid) | The Health Ministry publishes a set of guidelines for the prevention of COVID exposure by occupational risk services |
| 6/3/20 |  | The Spanish Episcopal Conference mandates prevention measures at Catholic churches |
| 7/3/20 |  | Madrid: closure of nursing homes for the elderly, following infections in two of them.  La Rioja: lockdown in Haro due to a concentration of cases |
| 8/3/20 | 674 cases and 10 deceased | Sports events, political party conferences and IWD demonstrations. In the nationwide meeting of the far-right party Vox, its General Secretary JOS was already infected and symptomatic (will test positive on 10/3/20). |
| 9/3/20 |  | **Moderate social-distancing measures at Madrid:** closure of kindergartens, schools and universities.  Catalonian government bans all events with >1,000 persons  The Basque government closes all schools in Vitoria and Labastida. |
| 10/3/20 |  | *Italy declares national lockdown*  Champion League Match between Valencia and Atlanta de Bérgamo in Valencia  **Moderate social-distancing measures in Spain:**  Ban on all air travel to/from Italy  Ban on all indoor activities with more than 1,000 persons at Madrid, La Rioja, Vitoria & Labastida  **Moderate social-distancing measures at Madrid, La Rioja and Vitoria,** including the closure of schools and universities. |
| 11/3/20 |  | *WHO declares COVID-19 outbreak an international pandemia*  Closure of national Congress and Senate, and regional parliaments of Madrid and Andalusia  3,000 Atlético de Madrid fans flew together for a Champions League match in Liverpool |
| 12/3/20 |  | **Moderate social-distancing mandated by all regional governments:** Nationwide closure of schools and universities. Confinement of four municipalities in Catalonia.  The central government launch a package of measures to mitigate the economic impact of the outbreak ([Real Decreto-ley 7/2020, de 12 de marzo](http://www.boe.es/buscar/act.php?id=BOE-A-2020-3580)). |
| 13/3/20 | WHO considers Europe the new epicenter of the pandemic. | Government announces **national lockdown**, pending on the decree’s approval.  Catalonia closes the regional borders. |
| 14/3/20 |  | **Nationwide lockdown:** [Real Decreto 463/2020](https://www.boe.es/eli/es/rd/2020/03/14/463/con) por el que se declara el estado de alarma para la gestión de la situación de crisis sanitaria ocasionada por el COVID-19 |
| 15/3/20 | Valencia FC acknowledges that its players and technical staff got infected on 19/2/20 |  |
| 16/3/20 |  | Ford Motor Co. closes factory in Valencia employing 5,4000 people, after three workers test positive for coronavirus |
| 17/3/20 |  | *EU restricts non-essential travel from/to its countries*  Spain closes all its land borders |
| 18/3/20 |  | Protocols for the [disinfection of spaces and surfaces in contact with suspected or confirmed COVID patients](https://www.comunidad.madrid/sites/default/files/doc/sanidad/samb/protocolo_desinfeccion_superficies_covid-19_180320.pdf) published by Madrid regional government |
| 19/3/20 | Italy overtakes China for the largest number of deaths from the pandemic |  |
| 21/3/20 |  | Government announces the purchase of 640,000 rapid tests. >350,000 tests have already been conducted. |
| 22/3/20 |  | Nationwide lockdown extended for another 15 days.  Opening of IFEMA’s temporary hospital, with 5,500 beds, in Madrid |
| 24/3/20 |  | Emergency Military Units enter retirement homes, where mortality was rocketing, to assist their elderly patients.  Madrid ice rink adapted as makeshift mortuary to cope with the high mortality. |
| 25/3/20 |  |  |
| 26/3/20 |  | Closure of hotels and short-stay accommodation (such as short-stay campsites or caravan parks) |
| 27/3/20 |  | Madrid launches emergency action plan at nursing homes, which accumulate >50% of Madrid’s mortality. |
| 29/3/20 |  | Strengthened nationwide lockdown: closure of the workplaces of companies that do not provide essential services |
| 1/4/20 | Spain surpasses 100,000 infections |  |
| 2/4/20 |  | Emergency Military Units asked to assist disinfecting 84 retirement homes in Catalonia, owing to the high mortality and infection rate |
| 3/4/20 | COVID basic reproduction number <1.0 for the first time in Spain. |  |

**Supplementary Table S2.** Parameter estimates of segmented regressions fitted on the total number of cases detected in Spain and the two autonomous regions hosting its two largest cities (Madrid and Catalonia) from 25/2/20 to 13/04/20, using the segmented and lme4 packages of R 3.6.3 (R Core Team 2020). Within this period, data series varies among variables and regions, since they start at the first day with >10 cases or >1 death. rDev: residual deviance. RL: relative likelihood.

**Supplementary Table S3.** Confidence intervals of the breakpoints estimated by segmented regressions on the total number of cases detected in Spain and the two autonomous regions hosting its two largest cities (Madrid and Catalonia) from 25/2/20 to 13/04/20, using the segmented and lme4 packages of R 3.6.3 (R Core Team 2020). Within this period, data series varies among variables and regions, since they start at the first day with >10 cases or >1 death. Only the results of the best model (i.e. the most parsimonious model with a significantly lower AIC) are presented.


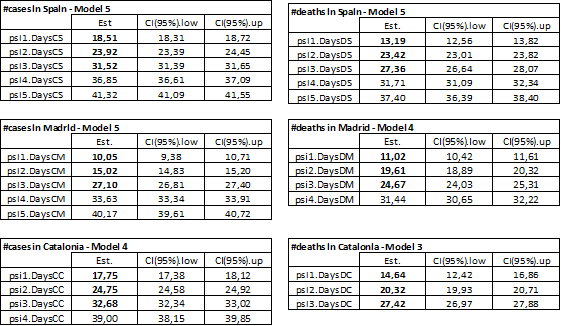


**Supplementary Table S4.** Dataset. CS: number of cases in Spain. DS: number of deaths in Spain. CM: number of cases in Madrid. DM: number of deaths in Madrid. CC: number of cases in Catalonia. DC: number of deaths in Catalonia.

**Supplementary Table S5.** Script of the breakpoint analysis based on R’s segmented procedure. The script was identical for all regions, except for the selection of vectors associated to the timeline, which varied slightly among datasets. CS: number of cases in Spain. DS: number of deaths in Spain. CM: number of cases in Madrid. DM: number of deaths in Madrid. CC: number of cases in Catalonia. DC: number of deaths in Catalonia.

library(segmented)

library(modelr)

# view the dataset and print a list of variables name

View(Dataset)

names(Dataset)

## Reference model, without breakpoints, using a poisson error distribution with a log link

RegModel.1 <- glm(Cases~Days.Cases, family=poisson(link = "log"), data=Dataset)

summary(RegModel.1)

with(RegModel.1, cbind(res.deviance = deviance, df = df.residual, p = pchisq(deviance, df.residual, lower.tail=FALSE)))

AIC0 <- AIC(RegModel.1)

BIC0 <- BIC(RegModel.1)

# plot fit

plot(RegModel.1, conf.level=0.95, shade=TRUE)

points(LnCases ~ Days.Cases , data = Dataset)

## testing for one breakpoint, based on the fit of the reference model

segmented1.mod <- segmented(RegModel.1, seg.Z = ~Days.Cases, psi=17)

summary(segmented1.mod)

with(segmented1.mod, cbind(res.deviance = deviance, df = df.residual, p = pchisq(deviance, df.residual, lower.tail=FALSE)))

AIC1 <- AIC(segmented1.mod)

BIC1 <- BIC(segmented1.mod)

# obtain estimators of the slopes and confident intervals of breakpoints

slope(segmented1.mod)

confint(segmented1.mod) #delta CI

# plot fit

plot(segmented1.mod, conf.level=0.95, shade=TRUE)

points(LnCases ~ Days.Cases , data = Dataset)

points(segmented1.mod, link=TRUE, col=2)

lines(segmented1.mod,col=2,pch=19,bottom=FALSE,lwd=2) #for the CI for the breakpointpoints

## testing for 2 breakpoints, by specifying the starting values (vector) for psi:

segmented2.mod<-segmented(RegModel.1,seg.Z=~Days.Cases,psi=c(11,22), control=seg.control(display=FALSE))

summary(segmented2.mod)

with(segmented2.mod, cbind(res.deviance = deviance, df = df.residual, p = pchisq(deviance, df.residual, lower.tail=FALSE)))

AIC2 <- AIC(segmented2.mod)

BIC2 <- BIC(segmented2.mod)

# obtaining estimators of the slopes and confident intervals of breakpoints

slope(segmented2.mod)

confint(segmented2.mod) #delta CI

# plot fit

plot(segmented2.mod, conf.level=0.95, shade=TRUE)

points(LnCases ~ Days.Cases , data = Dataset)

points(segmented2.mod, link=TRUE, col=2)

lines(segmented2.mod,col=2,pch=19,bottom=FALSE,lwd=2) #for the CI for the breakpointpoints

## testing for 2 breakpoints, by specifying only the *number* of breakpoints

segmented2b.mod<-segmented(RegModel.1,seg.Z=~Days.Cases, npsi=2, control=seg.control(display=FALSE))

summary(segmented2b.mod)

with(segmented2b.mod, cbind(res.deviance = deviance, df = df.residual, p = pchisq(deviance, df.residual, lower.tail=FALSE)))

AIC2b <- AIC(segmented2b.mod)

BIC2b <- BIC(segmented2b.mod)

# obtaining estimators of the slopes and confident intervals of breakpoints

slope(segmented2b.mod)

confint(segmented2b.mod) #delta CI

# plot fit

plot(segmented2b.mod, conf.level=0.95, shade=TRUE)

points(LnCases ~ Days.Cases , data = Dataset)

points(segmented2b.mod, link=TRUE, col=2)

lines(segmented2b.mod,col=2,pch=19,bottom=FALSE,lwd=2) #for the CI for the breakpointpoints

## testing for 3 breakpoints, by specifying the starting values (vector) for psi:

segmented3.mod<-segmented(RegModel.1,seg.Z=~Days.Cases,psi=c(11,22,33), control=seg.control(display=FALSE))

summary(segmented3.mod)

with(segmented3.mod, cbind(res.deviance = deviance, df = df.residual, p = pchisq(deviance, df.residual, lower.tail=FALSE)))

AIC3 <- AIC(segmented3.mod)

BIC3 <- BIC(segmented3.mod)

# obtaining estimators of the slopes and confident intervals of breakpoints

slope(segmented3.mod)

confint(segmented3.mod) #delta CI

# plot fit

plot(segmented3.mod, conf.level=0.95, shade=TRUE)

points(LnCases ~ Days.Cases , data = Dataset)

points(segmented3.mod, link=TRUE, col=2)

lines(segmented3.mod,col=2,pch=19,bottom=FALSE,lwd=2) #for the CI for the breakpointpoints

## testing for 3 breakpoints, by specifying just the *number* of breakpoints

segmented3b.mod<-segmented(RegModel.1,seg.Z=~Days.Cases, npsi=3, control=seg.control(display=FALSE))

summary(segmented3b.mod)

with(segmented3b.mod, cbind(res.deviance = deviance, df = df.residual, p = pchisq(deviance, df.residual, lower.tail=FALSE)))

AIC3b <- AIC(segmented3b.mod)

BIC3b <- BIC(segmented3b.mod)

# obtaining estimators of the slopes and confident intervals of breakpoints

slope(segmented3b.mod)

confint(segmented3b.mod) #delta CI

# plot fit

plot(segmented3b.mod, conf.level=0.95, shade=TRUE)

points(LnCases ~ Days.Cases , data = Dataset)

points(segmented3b.mod, link=TRUE, col=2)

lines(segmented3b.mod,col=2,pch=19,bottom=FALSE,lwd=2) #for the CI for the breakpointpoints

# testing for 4 breakpoints, by specifying starting values (vector) for psi:

segmented4.mod<-segmented(RegModel.1,seg.Z=~Days.Cases,psi=c(9,18,27,36), control=seg.control(display=FALSE))

summary(segmented4.mod)

slope(segmented4.mod)

with(segmented4.mod, cbind(res.deviance = deviance, df = df.residual, p = pchisq(deviance, df.residual, lower.tail=FALSE)))

AIC4 <- AIC(segmented4.mod)

BIC4 <- BIC(segmented4.mod)

# obtaining estimators of the slopes and confident intervals of breakpoints

slope(segmented4.mod)

confint(segmented4.mod) #delta CI

# plot fit

plot(segmented4.mod, conf.level=0.95, shade=TRUE)

points(LnCases ~ Days.Cases , data = Dataset)

points(segmented4.mod, link=TRUE, col=2)

lines(segmented4.mod,col=2,pch=19,bottom=FALSE,lwd=2) #for the CI for the breakpointpoints

# testing for 4 breakpoints, by specifying just the *number* of breakpoints

segmented4b.mod<-segmented(RegModel.1,seg.Z=~Days.Cases, npsi=4, control=seg.control(display=FALSE))

summary(segmented4b.mod)

with(segmented4b.mod, cbind(res.deviance = deviance, df = df.residual, p = pchisq(deviance, df.residual, lower.tail=FALSE)))

AIC4b <- AIC(segmented4b.mod)

BIC4b <- BIC(segmented4b.mod)

# obtaining estimators of the slopes and confident intervals of breakpoints

slope(segmented4b.mod)

confint(segmented4b.mod) #delta CI

# plot fit

plot(segmented4b.mod, conf.level=0.95, shade=TRUE)

points(LnCases ~ Days.Cases , data = Dataset)

points(segmented4b.mod, link=TRUE, col=2)

lines(segmented4b.mod,col=2,pch=19,bottom=FALSE,lwd=2) #for the CI for the breakpointpoints

# testing for 5 breakpoints, by specifying starting values (vector) for psi:

segmented5.mod<-segmented(RegModel.1,seg.Z=~Days.Cases,psi=c(7,14,21,28,35), control=seg.control(display=FALSE))

summary(segmented5.mod)

slope(segmented5.mod)

with(segmented5.mod, cbind(res.deviance = deviance, df = df.residual, p = pchisq(deviance, df.residual, lower.tail=FALSE)))

AIC5 <- AIC(segmented5.mod)

BIC5 <- BIC(segmented5.mod)

# obtaining estimators of the slopes and confident intervals of breakpoints

slope(segmented5.mod)

confint(segmented5.mod) #delta CI

# plot fit

plot(segmented5.mod, conf.level=0.95, shade=TRUE)

points(LnCases ~ Days.Cases , data = Dataset)

points(segmented5.mod, link=TRUE, col=2)

lines(segmented5.mod,col=2,pch=19,bottom=FALSE,lwd=2) #for the CI for the breakpointpoints

## Select best model

AIC <- c(AIC0, AIC1, AIC2, AIC2b, AIC3, AIC3b, AIC4, AIC4b, AIC5)

print(AIC)

min(AIC)

## plot best model with upper axis showing observation dates

plot(segmented5.mod, conf.level=0.95, shade=TRUE, axes=FALSE, xlab="Number of days", ylab="Ln(Number of cases in Madrid)", cex.lab=1.3, cex.at=x)

# draw an axis on the top

x <- c(0,7,14,21,28,35,42)

lbls <- c("29/2/20","7/3/20","14/3/20","21/320","28/3/20","4/4/20","11/4/20")

axis(3, at=x, labels=lbls, col.axis="black", las=1, tck=-.03)

axis(3, at=0:43, labels=FALSE, col.axis="black", las=1, tck=-.015)

# include observed values

points(LnCases ~ Days.Cases, data = Dataset)

# include breakpoints

points(segmented5.mod, link=TRUE, col=2)

# include the CI of the breakpointpoints

lines(segmented5.mod,col=2,pch=19,bottom=FALSE,lwd=2)

**Supplementary Figure S1.** Scheme of the key events related with the timeline of the COVID-19 pandemic in Spain, including the breaking points in the growth curves of the number of cases and deaths, at the time of detection (right column) and the estimated time of infection (left column). See Figure 2 for a graphical summary and Supplementary Table S1 for a detailed account of the timetable. Black numbers: change of slope at the breaking points of R’s segmented fits (all negative unless preceded by a + symbol). Red numbers: change of slope at the breaking points of R’s strucchange fits (all negative unless preceded by a + symbol). Blue blocks: clusters of breakpoints associated to decreases in slopes (growth rates). Orange blocks: cluster of breakpoints associated to increases in slopes (growth rates) or intercepts (one-day ‘jumps’ in values).

**Supplementary Figure S2.** Results of the simultaneous estimation of multiple breakpoints in segmented regressions fitted using the [breakpoints](https://www.rdocumentation.org/packages/strucchange/versions/1.5-2/topics/breakpoints) function of R’s [strucchange](https://www.rdocumentation.org/packages/strucchange/versions/1.5-1) package, on the total number of cases detected in Spain and the two autonomous regions hosting its two largest cities (Madrid and Catalonia) from 25/2/20 to 13/04/20. Within this period, data series varies among variables and regions, since they start at the first day with >10 cases or >1 death. Lines show the best fit, i.e. that with the lowest BIC (see also Supplementary Figure S3). Broken red lines indicate breaking points of the best fit.


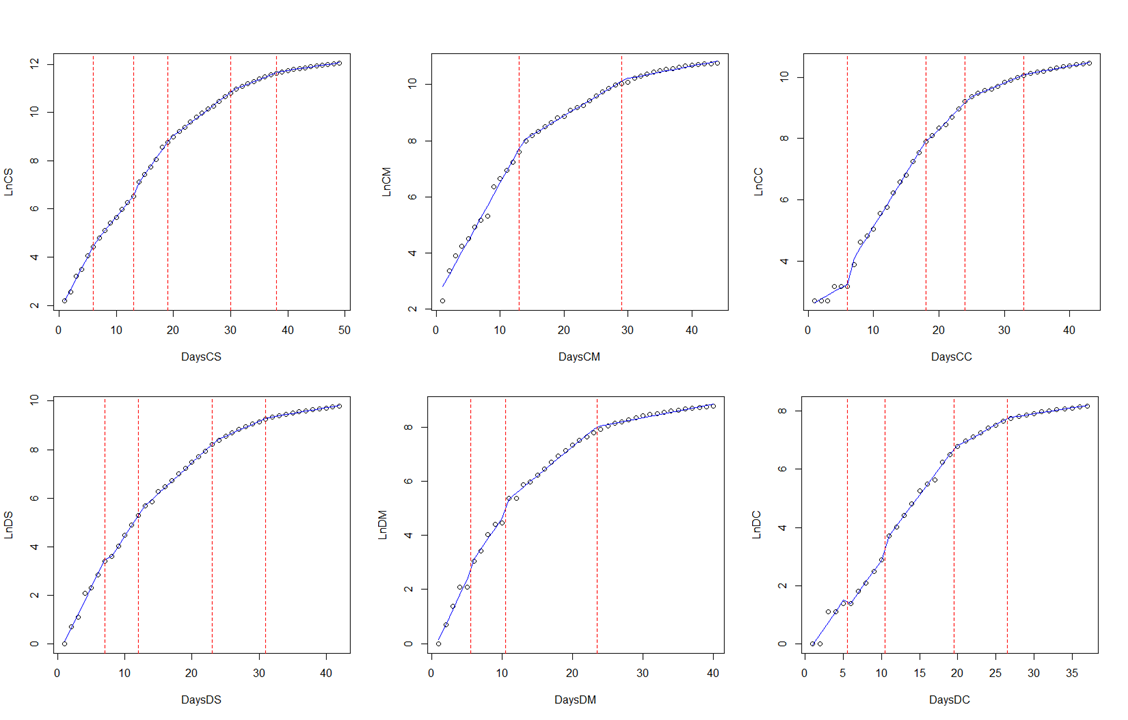


**Supplementary Figure S3.** Results of the simultaneous estimation of multiple breakpoints in segmented regressions fitted using the [breakpoints](https://www.rdocumentation.org/packages/strucchange/versions/1.5-2/topics/breakpoints) function of R’s [strucchange](https://www.rdocumentation.org/packages/strucchange/versions/1.5-1) package, on the total number of cases detected in Spain and the two autonomous regions hosting its two largest cities (Madrid and Catalonia) from 25/2/20 to 13/04/20. Graphs show two goodness-of-fit parameters, the Bayesian Information Criterion (BIC, black lines and dots) and Residual Sum of Squares (blue lines and symbols) of a set of eight different models ranging from zero to seven breakpoints. The models with the lowest BIC were taken as the best fits and correspond to the lines displayed in Supplementary Figure S3.


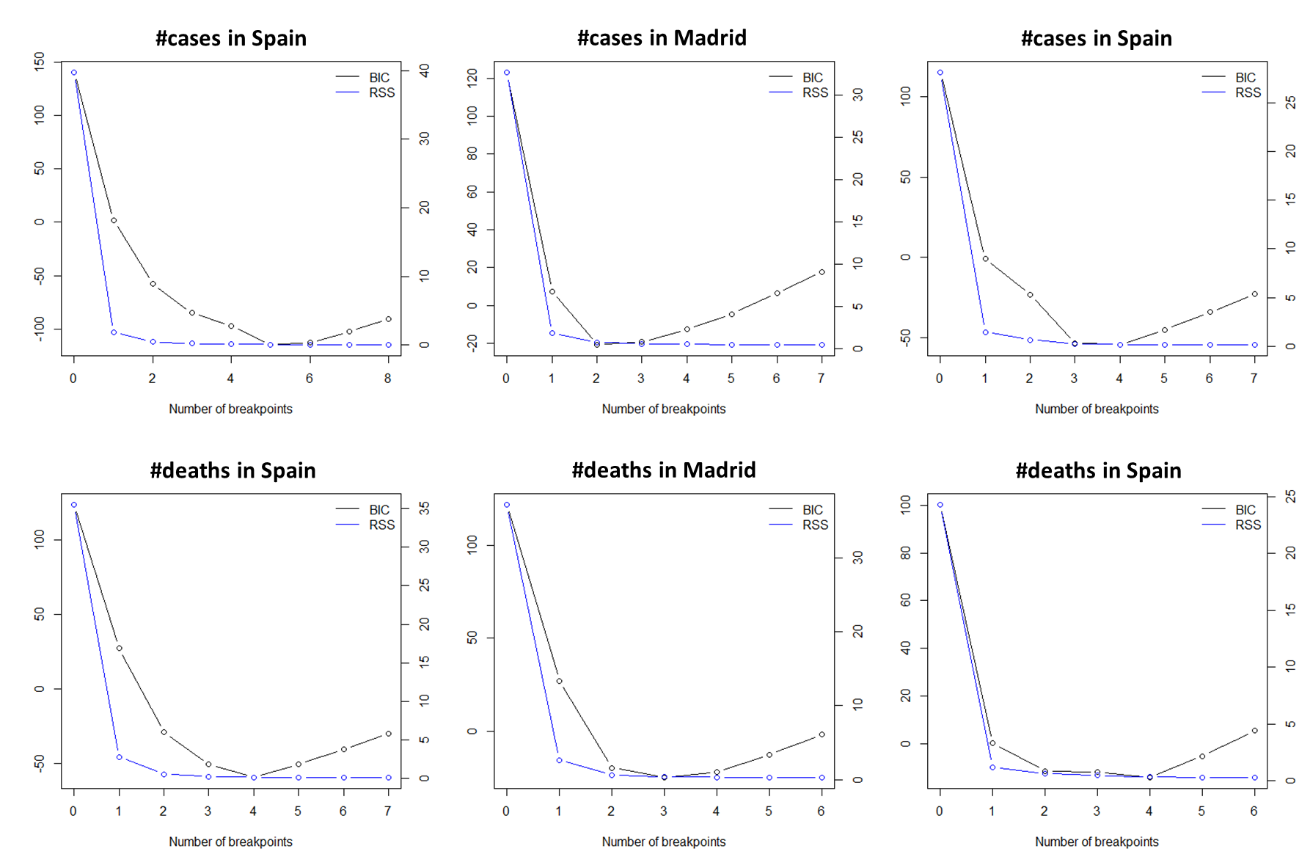


**Supplementary Figure S4.** Results of the simulations used to evaluate the detectability of a sudden infection bout, taking place on March 8^th^ 2020, through the segmented-regression analysis of the total number of cases detected in Madrid. Graphs show the different scenarios (upper left panel) and the best (i.e., lowest AIC) segmented regression fits of, respectively, the observed dataset (upper right panel) and the four five simulated datasets. Scenarios represent a single-day increase in number of cases of 25%, 50%, 75% and 100%, respectively. Red dots indicate breaking points of the best fit, with 95% confidence intervals (red lines). “R” indicates the breaking points of the observed dataset.


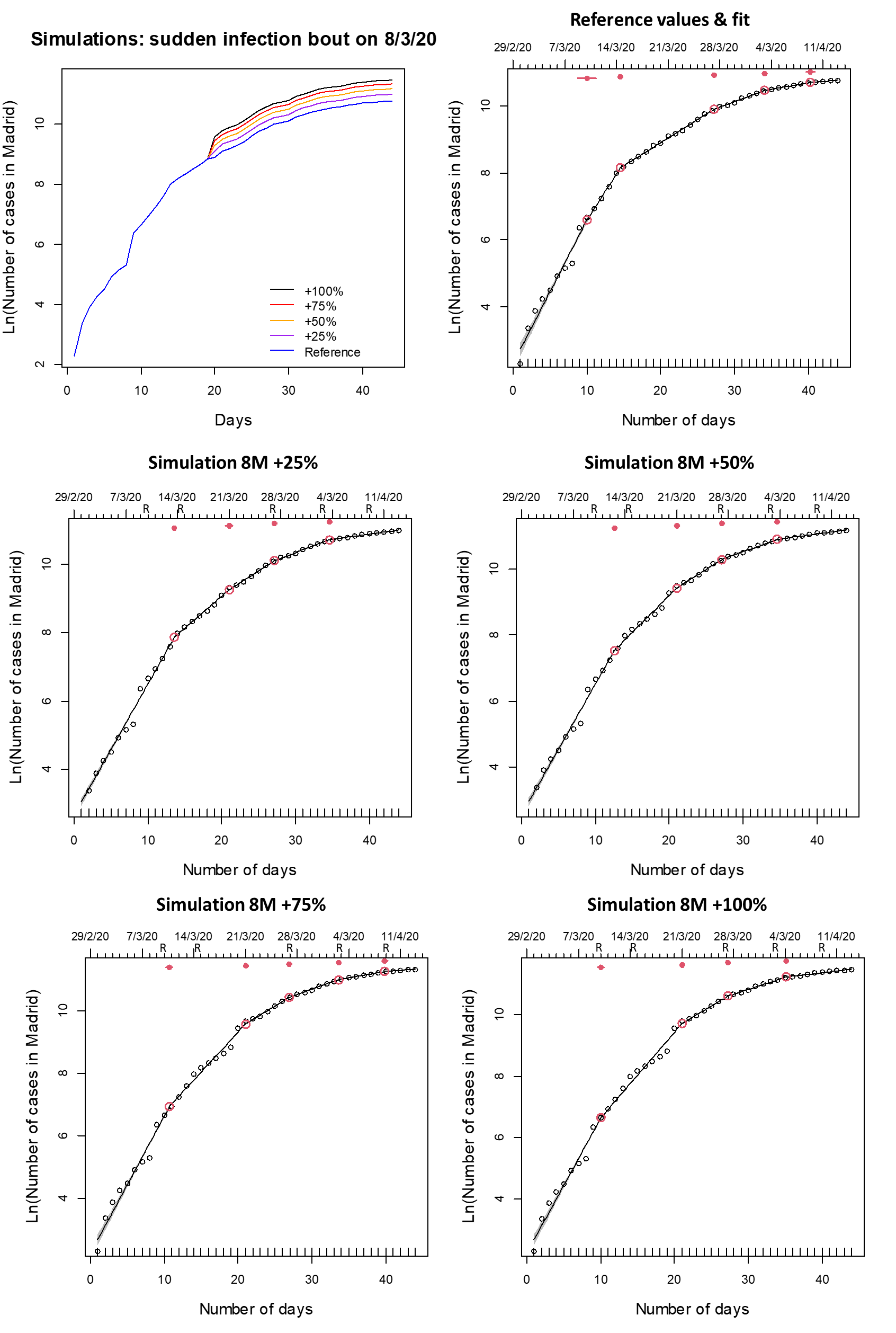


**Supplementary Figure S5.** Results of the simulations used to evaluate the detectability of a sustained increase in infection rate, taking place from March 8^th^ to March 15^th^ 2020, through the analysis of the total number of cases detected in Madrid. Graphs show the different scenarios (upper left panel) and the best (i.e., lowest AIC) segmented regression fits of, respectively, the observed dataset (upper right panel) and the four simulated datasets. Scenarios represent an increase in the daily rate of increase of the number of cases of 2.5%, 5%, 7.5% and 10%, respectively. Red dots indicate breaking points of the best fit, with 95% confidence intervals (red lines). “R” indicates the breaking points of the observed dataset.


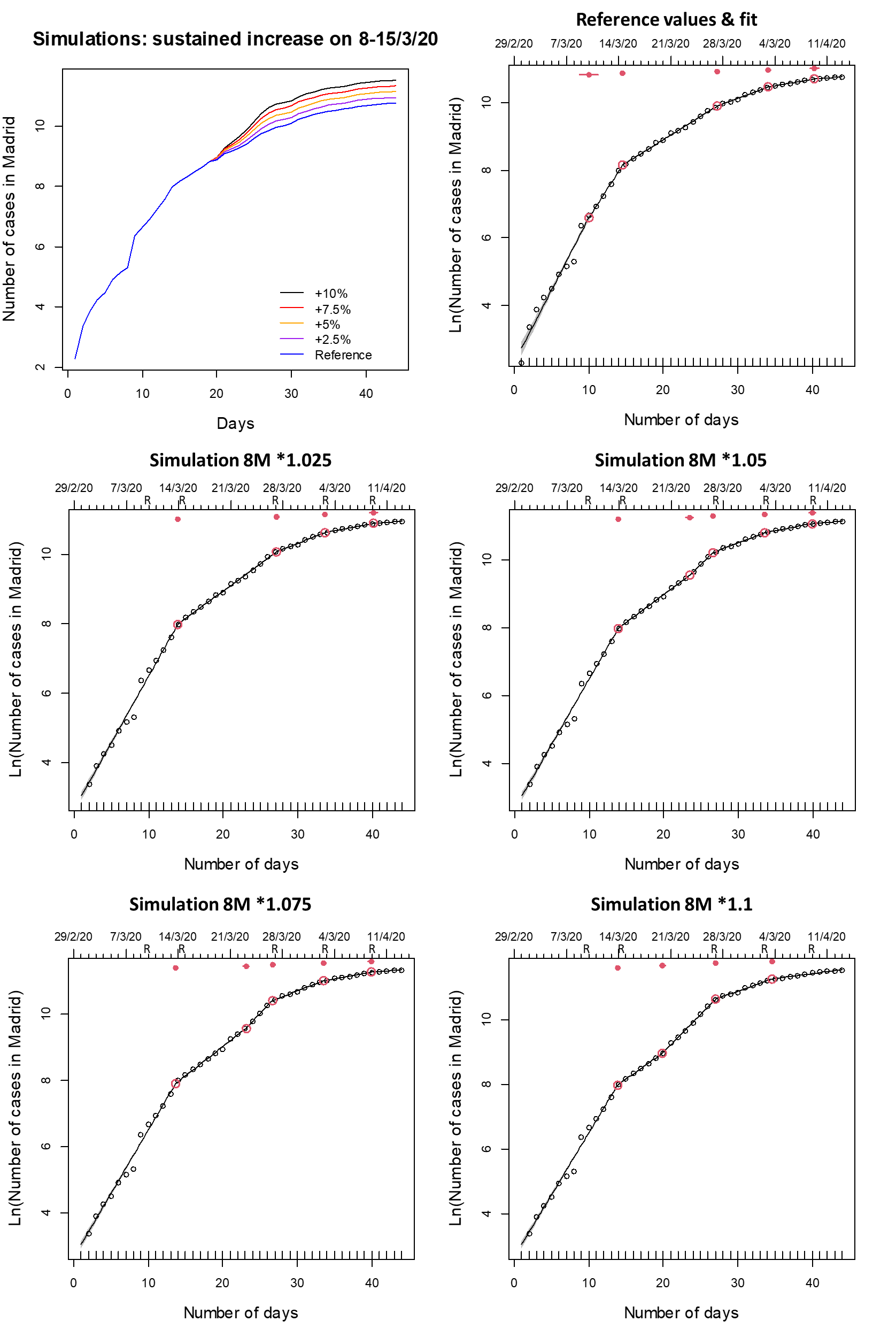


**Supplementary Figure S6.** Segmented regressions fitted on the Mobility Index (average of changes in mobility, as % of the baseline, in all categories except the residential locations) provided by the Google Mobility Report (2020) for Spain, Madrid region and Catalonia, from 25/2/20 to 13/04/20. Within this period, the data series varied analyzed slightly among variables and regions, to match the time period used for the analysis of the #cases and #deaths caused by Covid (see Figure 1, main text). Lines show the best fit, as specified in Table 1 (see also Tables S2-S3). Red dots indicate breaking points of the best fit, with 95% confidence intervals (red lines).


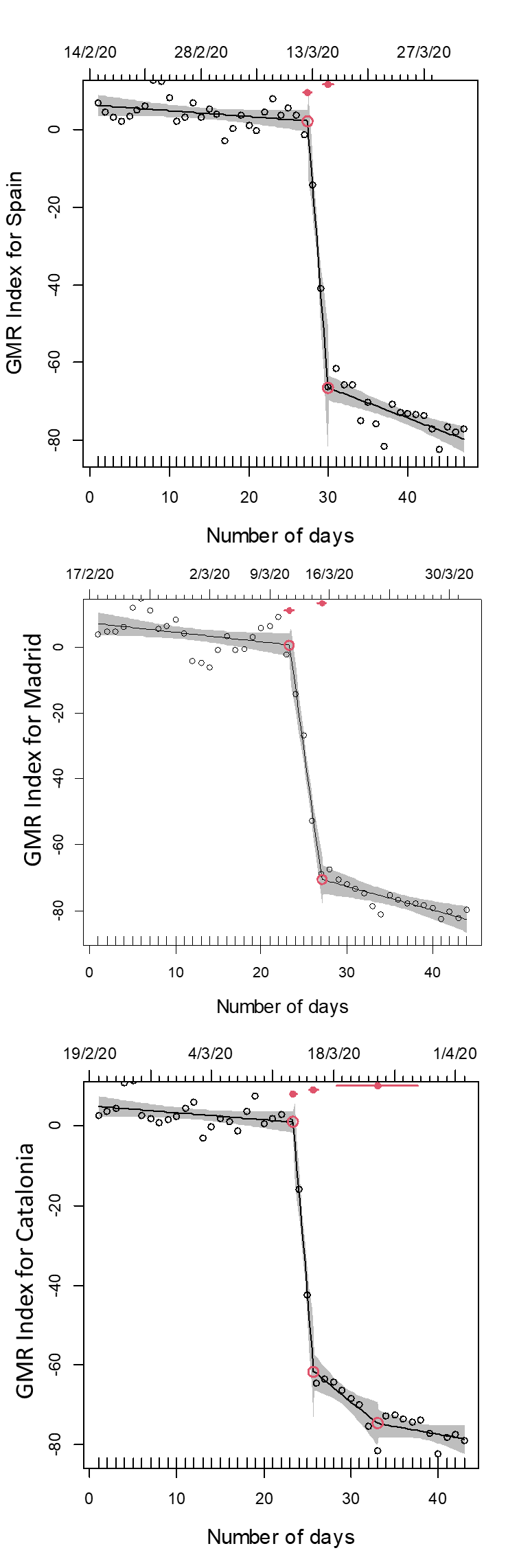

Supplement: Supplementary file 1 [file S0950268820002782sup001.docx]
